# A new version of the ANDSystem tool for automatic extraction of knowledge from scientific publications with expanded functionality for reconstruction of associative gene networks by considering tissue-specific gene expression

**DOI:** 10.1186/s12859-018-2567-6

**Published:** 2019-02-05

**Authors:** Vladimir A. Ivanisenko, Pavel S. Demenkov, Timofey V. Ivanisenko, Elena L. Mishchenko, Olga V. Saik

**Affiliations:** 1 Laboratory of Computer-Assisted Proteomics, Institute of Cytology and Genetics, Siberian Branch, Russian Academy of Sciences, Prospekt Lavrentyeva 10, Novosibirsk, 630090 Russia; 2 Novosibirsk State University, st. Pirogova 1, Novosibirsk, 630090 Russia

**Keywords:** ANDSystem, Associative gene networks, Tissue-specific gene expression, Extrinsic apoptotic signaling pathway, Automated extraction of knowledge, Text-mining

## Abstract

**Background:**

Consideration of tissue-specific gene expression in reconstruction and analysis of molecular genetic networks is necessary for a proper description of the processes occurring in a specified tissue. Currently, there are a number of computer systems that allow the user to reconstruct molecular-genetic networks using the data automatically extracted from the texts of scientific publications. Examples of such systems are STRING, Pathway Commons, MetaCore and Ingenuity. The MetaCore and Ingenuity systems permit taking into account tissue-specific gene expression during the reconstruction of gene networks. Previously, we developed the ANDSystem tool, which also provides an automated extraction of knowledge from scientific texts and allows the reconstruction of gene networks. The main difference between our system and other tools is in the different types of interactions between objects, which makes the ANDSystem complementary to existing well-known systems. However, previous versions of the ANDSystem did not contain any information on tissue-specific expression.

**Results:**

A new version of the ANDSystem has been developed. It offers the reconstruction of associative gene networks while taking into account the tissue-specific gene expression. The ANDSystem knowledge base features information on tissue-specific expression for 272 tissues. The system allows the reconstruction of combined gene networks, as well as performing the filtering of genes from such networks using the information on their tissue-specific expression. As an example of the application of such filtering, the gene network of the extrinsic apoptotic signaling pathway was analyzed. It was shown that considering different tissues can lead to changes in gene network structure, including changes in such indicators as betweenness centrality of vertices, clustering coefficient, network centralization, network density, etc.

**Conclusions:**

The consideration of tissue specificity can play an important role in the analysis of gene networks, in particular solving the problem of finding the most significant central genes. Thus, the new version of ANDSystem can be employed for a wide range of tasks related to biomedical studies of individual tissues. It is available at http://www-bionet.sscc.ru/and/cell/.

**Electronic supplementary material:**

The online version of this article (10.1186/s12859-018-2567-6) contains supplementary material, which is available to authorized users.

## Background

The rapid growth of data in the field of biology and biomedicine makes almost impossible for the researcher a complete analysis of this information without the use of automated text-mining tools. It should be especially noted that scientific publications are the main source of information for the vast majority of molecular genetic databases, which together contain only a part of the verified facts about the molecular interactions presented in publications. At the moment, the number of scientific publications (according to the PubMed database) and the number of international patents exceeds 28 million and 40 million, respectively, which allows regarding them as large text data. In fact, today, owing to the information explosion, we have a paradoxical situation in the biological sciences, where part of new knowledge, presented in publications, does not have a real opportunity to reach most researchers. One of the solutions to this is the development of computer tools that implement a full cycle of knowledge engineering and knowledge management, including an automated extraction of knowledge from scientific publications, patents and databases, formalization and accumulation of extracted data, and providing such data to end-users to deal with fundamental and practical tasks [1].

In particular, the integration of information in knowledge bases with its further analysis can allow the generation of new hypotheses and obtaining novel knowledge about complex processes or mechanisms based on information about the individual subsystems that are part of such processes. This allows the reconstruction of the molecular genetic mechanisms of the functioning of living systems, which is a necessary condition for study of almost any important task in modern biology and biomedicine, including the search for drug targets, an assessment of the potential efficacy and toxicity of new drugs in pre-clinical trials, identification of biomarker molecules to create effective diagnostic systems, the search for candidate genes for genotyping, etc. [2].

Thus, the methods of automated retrieval of information from unstructured textual data (text-mining) have been actively developed, and, in particular, such methods are popular in the fields of biology, biomedicine and various medical applications, including support for clinical decisions [3, 4], systems and integrative biology [5], curation of biological/biomedical databases [6], and pharmacovigilance inspections [7]. Modern text-mining techniques allow performance of an automated analysis of a wide range of information sources, including full-texts of scientific publications, patents and abstracts of articles [8, 9], as well as electronic health records of patients [10] and health-related data from social networks [7].

An automated recognition of the names of biological entities in natural language texts is one of the primary tasks of any text-mining-based research [11]. The aim of this task is to identify the names of objects of a specific type (proteins, genes, drugs, etc.) within the raw text. The solution of the name-recognition task is associated with a number of issues, primarily caused by the incompleteness of existing dictionaries of object names, as well as with the lack of universal rules for the naming of newly discovered objects. Additionally, challenges emanate from the high synonymy of biological objects along with different stylistic features often used by authors, including anaphora, epiphora, coreferent mentions, etc. The existing name-recognition approaches can be classified into three main categories: methods based on the use of dictionaries, rules-based methods, and machine-learning methods [11]. The ANDSystem tool combines rules-based and dictionaries-based methods [12].

Methods for the retrieval of information on the interactions between biological objects can also be divided into three main groups: (1) methods based on the statistically significant values of co-occurrence of object names in texts; (2) methods based on rules; and (3) methods based on machine-learning approaches. The main advantage of the first approach is its ease of implementation and robust completeness of the search, but at the same time, its accuracy is not high [13]. Moreover, such an approach does not allow detection of recently discovered interactions, information about which is not reflected in a large number of articles; also, this approach cannot be employed to determine specific parameters of interactions, such as the type of interaction or its direction.

Rules-based methods allow achievement of a high level of accuracy of information retrieval but, at the same time, have relatively low completeness values [14]. An alternative approach to automated information retrieval that does not require the use of manually created rules is machine-learning methods, which have been widely utilized in recent years. Examples of such methods are the naive Bayesian classifier, decision trees, conditional random fields [15], and structured support vector machines [16], as well as deep-learning algorithms based on neural networks [17]. In turn, these methods can be divided into three large categories [14]: 1) learning with the use of labelled data (supervised learning); (2) learning with the use of unlabeled data (semi-supervised learning); and (3) hybrid methods based on integrated training schemes. The “supervised learning” methods commonly require large corpora of textual data with mapped interactions. At the present time, a second group of machine-learning methods is actively developing, and, among them, special attention is being paid to those based on the use of external knowledge bases for training, the so-called methods with remote training. Such methods combine the advantages of both “supervised learning” and “unsupervised learning” [18]. An approach of this kind assumes that any sentence where a pair of objects from the base of knowledge is mentioned most likely describes the interaction between these objects. To reduce errors associated with the fact that certain sentences from a positive training set may feature pairs of objects, but do not describe their interactions, various approaches have been proposed, including multivariate training [19–21]. These approaches have proven themselves to perform really well with respect to tasks of identifying protein-protein interactions, gene-disease associations, and analysis of catalytic reactions [13, 22–26].

The most convenient form of representing extracted knowledge in the field of biology and biomedicine is semantic networks, where nodes correspond to biological objects, and edges correspond to interactions between them. The biological objects are molecular-genetic entities (genes, proteins, metabolites, drugs, microRNAs), biological processes, diseases, etc. The interactions between objects are determined by molecular, physical, chemical (protein-protein interactions, catalytic reactions), regulatory (activation, suppression, etc.), and associative (co-occurrence of object names in one sentence or document) relationships. The types of objects used and the relationships between them are specified by the domain ontology, specific for each computer system. On the basis of such ontologies, the molecular, cellular, and physiological mechanisms of functioning of living organisms under normal and pathological conditions can be described and visualized.

Among the computer programs that use text-mining methods for automated extraction of knowledge, special attention should be paid to software and information systems implementing a so-called full cycle of knowledge engineering, including knowledge extraction, integration, and presentation of data to the end user. The well-known examples of such systems are STRING (http://string-db.com), Coremine (https://www.coremine.com/), Pathway Commons (https://www.pathwaycommons.org/), MetaCore (https://clarivate.com/products/metacore/), and Ingenuity (https://www.qiagenbioinformatics.com/products/ingenuity-pathway-analysis/). All the extracted information is automatically stored in special knowledge bases of such systems, while the data representation takes place through the execution of user queries. Moreover, it is often the case that such systems include various statistical methods, for example, the prioritization of genes, the identification of overrepresented processes or diseases, etc., permitting the user to perform an analysis and interpretation of experimental data. It should be especially noted that the depth and detail of the knowledge representation in such systems is determined by the terms and concepts of the used ontology. In particular, the Ingenuity system makes use of seven object types (proteins, genes, complexes, cells, tissues, drugs, and diseases), the relationships between which are described by the following types of interactions: transcriptional regulation, miRNA-mRNA target, phosphorylation cascades, protein-protein, or protein-DNA interactions.

The use of such ontologies facilitates dealing with a wide range of problems related to analysis and visualization of disease mechanisms, gene expression, as well as proteomics and metabolomics data analysis. However, their narrow focus leads to the loss of information owing to the limitations in the descriptions of such ontologies and provides modern systems with the ability to extract just a small part of the knowledge presented in the texts of scientific publications [27]. For example, with Ingenuity and other computer systems, diseases are presented by a kind of generalized object that do not take into account various pathological conditions and dysfunctions, which cannot be considered independent diseases or their symptoms. At the same time, the number of references to such terms in the scientific literature, according to our estimates, can be in the hundreds of thousands. In particular, scientific articles contain a vast amount of information on the characterization of such dysfunctions as biomarkers, predictors, and risk factors for diseases. Huge amounts of information remain unused by the existing systems, including, for example, environmental factors which also are risk factors for many diseases.

Earlier, we developed the ANDSystem tool, a software and information system based on the syntactic-semantic rules and designed for automated extraction of medical and biological knowledge from scientific publications [12, 28, 29]. The original ontology of the ANDSystem provides a highly detailed description of the subject area. In particular, the distinctive characteristic of the ANDSystem is the fact that all interactions are subdivided into organisms and cell lines. Unlike most existing systems, proteins and genes are treated by the ANDSystem as separate entities, and catalytic reactions can be represented by an enzyme, a substrate, and a product, potentially including many participants.

The knowledge base of ANDSystem, created on the basis of the automated analysis of more than 25 million of texts from PubMed abstracts and dozens of biological and biomedical factual databases, is an unique resource featuring formalized information on more than 20 million interactions of various types (more than 25 types) between molecular genetic objects (proteins, genes, metabolites, microRNAs), biological processes, phenotypic signs, drugs and their side effects, and diseases including: physical interactions with forming of molecular complexes (protein/protein, protein/DNA, metabolite/protein); catalytic reactions and proteolytic events involving the substrate/enzyme/product, as well as substrate/product transformation events in the case of complex reactions where there is no description of the involved enzymes; regulatory interactions, divided into positive and negative regulation of gene expression, function/activity, transport, and protein stability, involving proteins, metabolites and drugs, regulation of protein translation involving miRNAs, regulation of biological processes and phenotypic traits involving proteins, metabolites and drugs; associative interactions of genes, proteins, metabolites, biological processes, phenotypic traits with diseases, etc. Each interaction in ANDSystem is characterized by its participants, the type and direction, and also by the organism and cell in which it occurred according to literature sources and external databases. It should be noted that, unlike the similar tools, genes and proteins within ANDSystem are treated as separate objects linked by a directed type of interaction (gene- > protein) - expression, and are classified by individual organisms. Furthermore, every interaction has a cell type attribute in which events related to this interaction were observed. The developed system surpasses its well-known analogues by the number of types of interactions and object types.

Regarding the use of ANDSystem, a number of scientific studies have been carried out, in particular an analysis of the data of high-throughput proteomic experiments related to the study of Helicobacter pylori and their contribution to the development of gastritis and gastric tumors [30]; the study of the proteomic profile of the urine of a healthy person in normal conditions and under the influence of space-flight factors [31, 32]; an analysis of the tissue-specific knockout effect of genes and a search for potential drug targets [33]; and the identification of new regulatory molecular genetic mechanisms in the life cycle of the hepatitis C virus [34]. The application of ANDSystem to the analysis of comorbid diseases has allowed predicting new molecular genetic mechanisms of comorbidity of asthma and hypertension [35], as well as dystropy (reverse comorbidity) that determined the relationship between asthma and tuberculosis [36]; to study the molecular mechanisms of the comorbid relationship between pre-eclampsia, diabetes (diabetes mellitus and gestational diabetes), and obesity [37]; to investigate the molecular interactions of glaucoma with more than 20 comorbid diseases [38], etc. Based on the ANDSystem knowledge base, a number of software tools for analysis of experimental gene sets have been developed, in particular, a web-based FunGeneNet program that performs automated reconstruction of the associative gene network for the user-specified gene set and identifies genes involved in a greater number of interactions than can be expected according to random reasons [39]. In addition, the NACE tool employs the ANDSystem knowledge base to evaluate the effectiveness of potential signaling in gene networks [40]. Using the ANDSystem technology, we developed the *Solanum TUBEROSUM* knowledge base [41, 42], which is a computer platform for the complex intellectual processing of big data in the field of potato growing providing: 1) automated analysis of texts of scientific publications and factual databases with extraction of knowledge about genetics, markers, breeding, seed production, diagnostics of pathogens, protective equipment, and potato-storage technologies; 2) formalized representation of the extracted information in the knowledge base; 3) user access to this data; and 4) analysis and visualization of user query results. The ontology of the *Solanum TUBEROSUM* knowledge base contains dictionaries of molecular genetic objects (proteins, genes, metabolites, microRNAs, biomarkers, etc.), potato varieties and their phenotypic traits, potato diseases and pests, biotic and abiotic environmental factors, agrobiotechnology of cultivation, and technologies of potato processing and storage.

Consideration of tissue-specific gene expression in the reconstruction of gene networks is a necessary condition for describing the processes taking place in the cells of this tissue. The ability to filter genes in gene networks by the level of their expression is realized in Ingenuity and some other systems, but absent in ANDSystem. As gene networks reconstructed with ANDSystem differ from similar networks created with other systems, the inclusion of information regarding tissue-specific gene expression in ANDSystem becomes a timely task. Thus, our paper is dedicated to the new version of ANDSystem, providing the reconstruction of tissue-specific gene networks. For this, we expanded ANDSystem with the data describing the expression of human genes in 272 different tissues, extracted from the Bgee database [43]. With regards to the example of the associative gene network of extrinsic apoptotic signaling pathway, it was shown that it was possible to filter the genes by their tissue-specific expression effects, such as network characteristics like the distribution of centrality of the vertices, the clustering coefficient, network centralization, the number of shortest paths, etc. Such effects can determine the difference in the functioning of the same processes in varying tissues. A new version of ANDSystem is available at http://www-bionet.sscc.ru/and/cell/.

## Methods

**Fig. 1:**
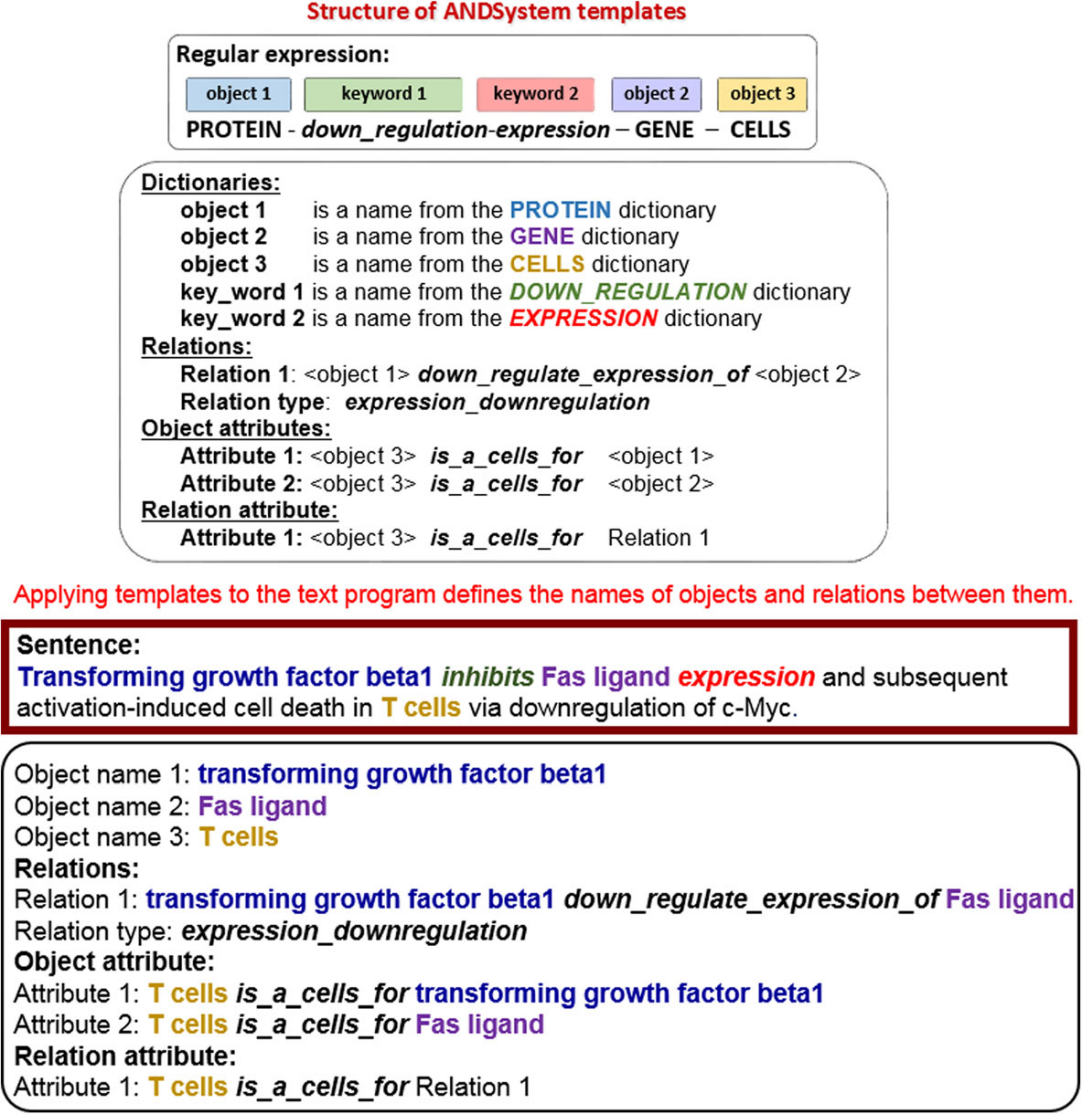
An example of semantic-linguistic template from ANDSystem and result of its application for analysis of a sentence from Genestier et al., 1999 [62]

The text-mining module of ANDSystem performs all the necessary preprocessing of textual data, including conversion of input text into the ANDSystem format, text segmentation, normalization, morphological and syntactic analysis, mapping of the named entities (object names), etc. The mapping is carried out by ANDSystem utilizing a complex dictionary-based algorithm. Then, the semantic-linguistic templates are applied to such preprocessed texts for the automated retrieval of information regarding interactions between the mapped objects. Next, all retrieved interactions are classified by organisms and cell lines using the special patterns, which perform identification of cells and species within the analyzed text. Semantic-linguistic templates of the ANDSystem have a complex structure that can be distinguished into two separate parts, where a first part is presented by syntactic relationships between the names of objects and keywords in the sentence, while a second is the semantic links between the objects (Fig. 1).

Data extracted using the templates are automatically stored in the ANDCell knowledge base, which is a module of ANDSystem. The analysis of publication texts and the filling of the ANDCell knowledge base are carried out on the server side of the system. Also, ANDSystem includes the ANDVisio program, which is a graphical user interface for the ANDCell database. It allows users to conduct reconstruction, expansion, graphic visualization, data filtering, as well as laying out of the gene network graph. The filtering in ANDVisio can be completed by object type, interaction type, data source, and organism [28].

The following structural characteristics of associative gene networks are calculated in ANDVisio:

- Number of nodes [44].
- Clustering coefficient [45]. The clustering coefficient of a given node is the probability that the two nearest neighbors of this node are also the nearest neighbors of each other. The clustering coefficient is the degree that determines how much the nodes are tending to cluster.
- Number of connected components [46] is a number of subgraphs in which any two vertices are connected to each other by paths, and which are connected to no additional vertices in the graph.
- Network centralization [47] that is a network-level, macrostructural measure that quantifies how ‘dispersed’ the centralities of the nodes are. When the measure is large, it means that few nodes are highly central, and the remaining occupy much fewer central positions in the network. Conversely, if network centralization is low, this means that the network is populated by nodes which occupy similarly central positions.
- Number of shortest paths [46] – the total number of shortest paths between all pairs of vertices in the graph. The shortest path is a path between two vertices in a graph such that the sum of the weights of its constituent edges is minimized. If the weights of edges are not specified, they are considered equal to 1.
- Average number of neighbors [44, 46].
- Network density [46] that describes the portion of the potential connections in a network that are actual connections. A “potential connection” is a connection that could possibly exist between two “nodes”, regardless of whether or not it actually does, e.g., this person could know that person; this computer could connect to that one. Whether or not they do connect is irrelevant when one is referring to a potential connection. In contrast, an “actual connection” is one that actually exists, e.g., this person does know that person; this computer is connected to that one.
- Network heterogeneity [48] that reflects the tendency of a network to contain hub nodes.

Bgee database (https://bgee.org/) contained information on gene expression patterns in multiple animal species obtained from different types of experiments, including RNA sequencing, Affymetrix microarray analysis, in situ hybridization, and expressed sequence tag (EST) surveys [43]. Bgee provides information on the presence/absence of gene expression under normal conditions (e.g., no gene knockout, no treatment, no disease). The data on tissue-specific gene expression were extracted from the Bgee database and used in ANDSystem “as is”.

The evaluation of the statistical significance of differences in the structural characteristics of associative gene networks was carried out using the generation of pseudo-random gene networks. For each associative gene network, including a combined and five tissue-specific networks, 1000 pseudo-random networks were reconstructed. For generating of pseudo-random networks, the number of vertices was set equal to the number of vertices in the original network. Vertices were randomly selected from all human genes presented in ANDSystem, the interactions between the selected vertices were automatically reconstructed using ANDSystem as well. For each generated pseudo-random network, seven indicators of structural characteristics were calculated. Then all possible pairs of a combined network with tissue-specific networks were considered. For each such pair, seven indicators were calculated, representing the difference between the values of structural characteristics of the networks in this pair. For each indicator, a distribution by 1000 values was built, according to the number of the generated pseudo-random networks. Additionally, five pairs were considered, each including an original combined and a tissue-specific network. The statistical significance of the indicator difference for pairs of original networks and pairs of pseudo-random networks was estimated based on the indicator values for the original pairs falling below the 5% or above 95% quantiles on the distribution density graph for pseudo-random pairs.

## Results and discussion

### Extension of ANDSystem

**Fig. 2:**
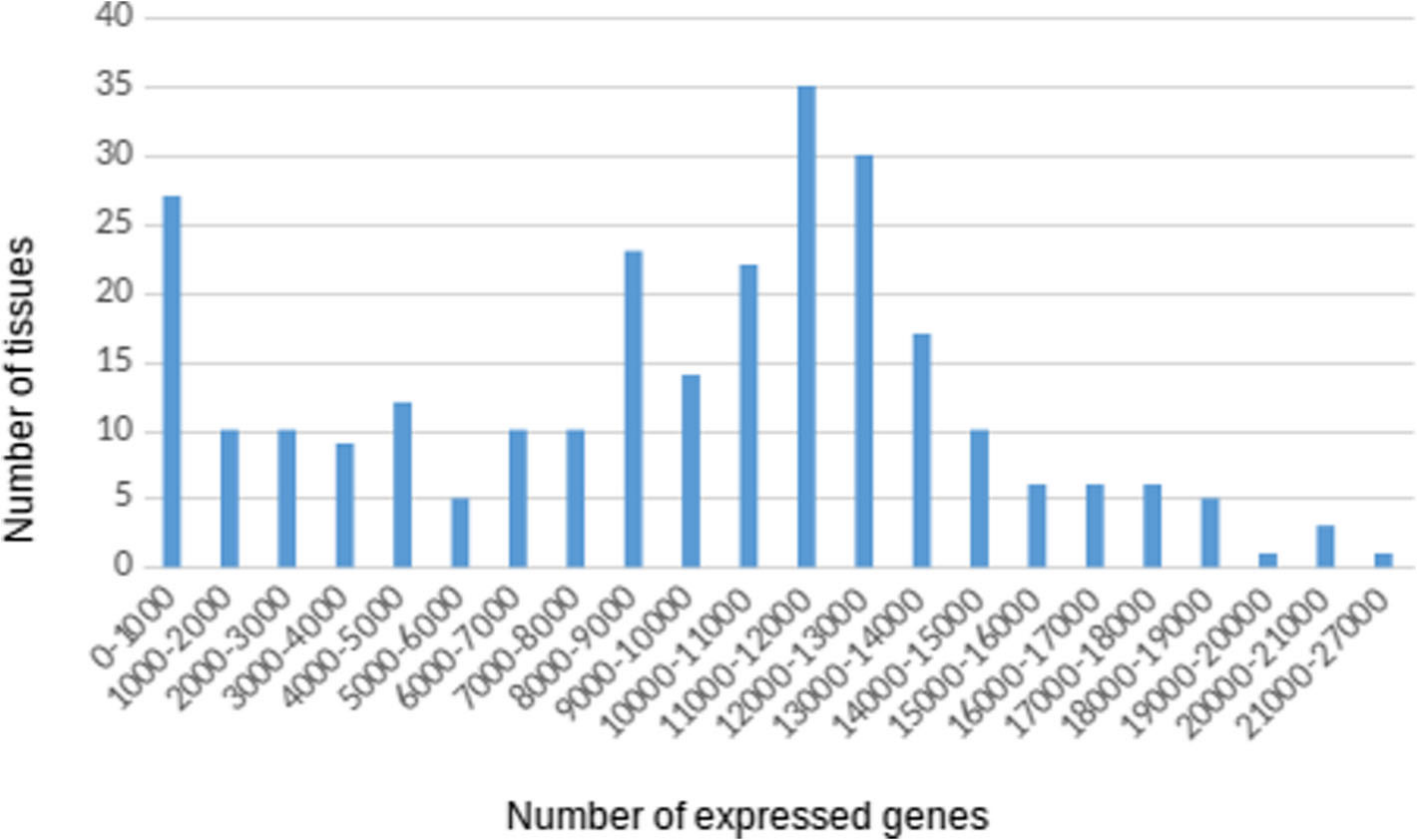
The distribution of the number of tissues depending on the number of genes expressed in them

Data on the expression of human genes in 272 tissues were loaded from the Bgee database [43] into ANDSystem. Figure 2 depicts the dependence of the number of tissues on the number of genes expressed in them. From the figure, it can be seen that roughly 11,000 to 12,000 genes are expressed in the majority of tissues. The highest number of genes (26,363) was expressed in the testis. More than 20,000 genes were expressed in the female gonads and prostate gland. It should be noted that for the 12 tissues, information on expression was presented only for less than 100 genes. Meanwhile, for four tissues (corpus striatum, osseus labyrinth vestibule, parathyroid gland and seminiferous tubule of testis), the number of expressed genes appeared to be less than 10.

**Fig. 3:**
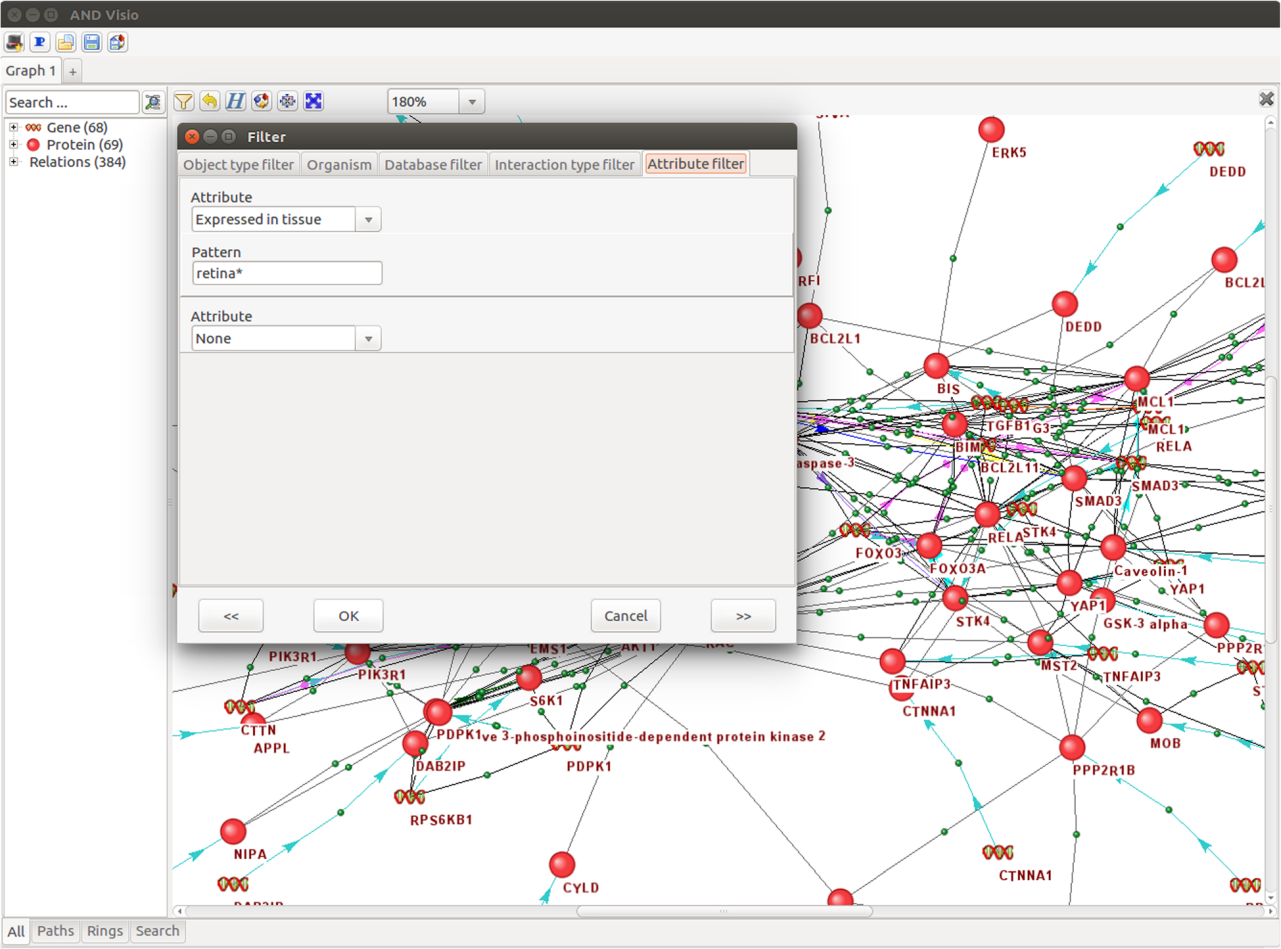
An example of tissue selection in the ANDVisio program for the filtering of genes by their tissue-specific expression

For filtering genes by their expression in a given tissue, a new filter was added to ANDVisio (Fig. 3). To filter, in the “Filter” window of the ANDVisio visualizer, the user must click on the “Attribute filter” tab, then select “Expressed in tissue” from the top drop-down list entitled “Attribute”, and then type the name of the tissue of interest in the “Pattern” field. Moreover, ANDVisio allows searching for the names of tissues by their partial matching, and for this, the user needs to add an asterisk character to the end of the name.

### Analysis of the associative gene network of apoptosis

Apoptosis is a form of programmed cell death. Disruptions of apoptosis are associated with many diseases, including cancer, neurodegenerative, autoimmune, inflammatory diseases, and others [49–53]. There are two main apoptosis pathways: the extrinsic apoptotic pathway mediated by cell death receptors and the mitochondrial pathway [54, 55]. The study of tissue specificity of apoptotic signal transduction, in particular, was performed by Yoshinori Otsuki, 2004 [56]. Of particular interest to researchers is the evaluation of tissue-specificity for the extrinsic apoptotic pathway [57, 58]. Using the new functionality of ANDSystem, it was interesting for us to perform the filtering of genes according to their tissue-specific expression and analyze the structural features of the filtered associative gene network of the extrinsic apoptotic signaling pathway. The associative gene network of extrinsic apoptosis was reconstructed automatically with ANDSystem. A set of human genes from the “extrinsic apoptotic signaling pathway” Gene Ontology biological process (GO: 0097191) was used as input data. In total, 220 genes were involved in this process. A list of these genes was obtained using AmiGO 2 (http://amigo.geneontology.org/amigo).

Lists of genes involved in the extrinsic apoptotic signaling pathway, which are expressed in various tissues, are available in Additional file 1: Table S1. For the analysis, five tissues were selected, including the lymph node, endometrium, embryo, retina, and substantia nigra. It is known that apoptosis actively occurs in the lymph node, endometrium, and embryo tissues in the norm [56]. At the same time, the activation of apoptosis in the retina and substantia nigra often leads to pathologies [59–61]. In this regard, it was noteworthy evaluating the differences in the structural organization of associative gene networks of apoptosis.

For each of these tissues, a tissue-specific associative gene network of extrinsic apoptotic signaling pathway was reconstructed, which included only genes expressed in a given tissue, then the structural characteristics of all the reconstructed networks were calculated (Table 1). Further, the combined network was generated, which is an associative gene network of an extrinsic apoptotic signaling pathway reconstructed without applying tissue-specific expression filters. From Table 1, it can be observed that the largest number of vertices among the five analyzed tissue networks was in the lymph node network, which had only 38 vertices less than in the combined network. The smallest network was that reconstructed for the retina. It was interesting to note that while the size of the embryo network was not the largest, it had the maximum clustering coefficient. The maximum value of the number of connected components was for the combined network, the largest network. At the same time, even though the retina network was the smallest, the value of this indicator for it exceeded the values for the endometrium and substantia nigra networks. It can also be highlighted that the network reconstructed for the substantia nigra had the highest network density value, while the lowest value of this parameter was for the embryo network. The network centralization, number of shortest paths, average number of neighbors, and network heterogeneity parameters correlated with the number of vertices in the networks.

**Table 1:** The structural characteristics of the reconstructed associative gene networks of the extrinsic apoptotic signaling pathway

Name	Combined network	Lymph node	Endometrium	Embryo	Retina	Substantia nigra
Number of nodes	441	403	389	307	175	345
Clustering coefficient	0,393,954	0,400,993	0,406,731	0,40,788	0,335,677^a^	0,403,556
Number of connected components	22	18	14^a^	18^a^	16^a^	14^a^
Network centralization	0,262,094	0,265,214	0,216,362^a^	0,203,354^a^	0,182,878^a^	0,212,608^a^
Number of shortest paths	1,381,738	1149342^a^	1125256^a^	551226^a^	82194^a^	793364^a^
Average number of neighbors	13,20,181	12,91,315^a^	12,48,329^a^	9179153^a^	5542857^a^	11,28,696^a^
Network density	0,030004	0,032122^a^	0,032173^a^	0,029997	0,031856	0,032811^a^
Network heterogeneity	120,3538	112,1453	105,4192^a^	78,93,359^a^	45,57,004^a^	94,84,783^a^

^a^ statistically significant differences from the combined network with *p*-value< 0.05

All five tissue-specific associative gene networks were statistically significantly different from the combined network by values of Number of shortest paths and Average number of neighbors. The Clustering coefficient was the least informative among all the other structural characteristics, because, according to this indicator, only one retina gene network had statistically significant differences from the combined network.

**Fig. 4:**
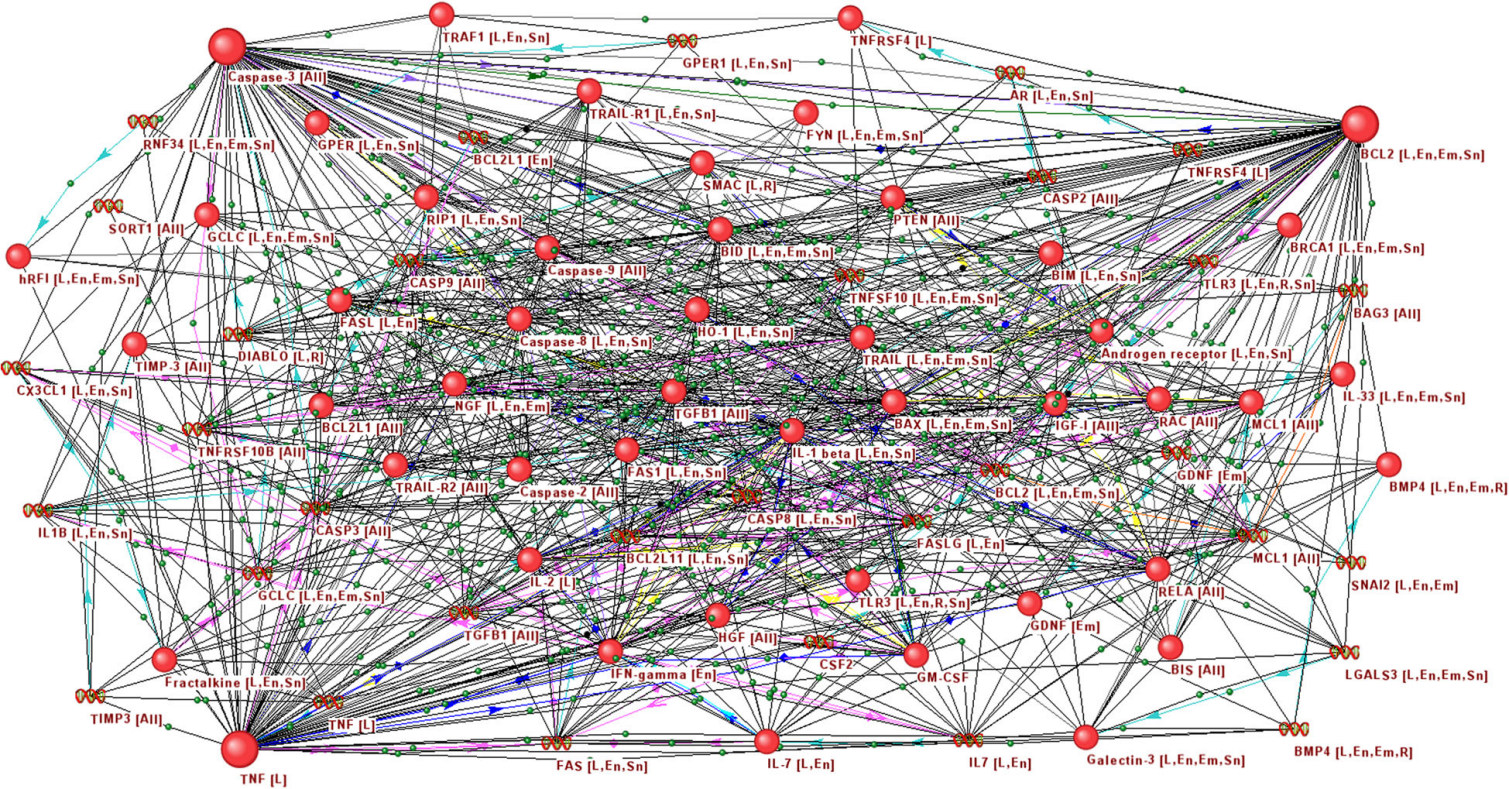
Associative gene network describing interactions between three central genes/proteins of the extrinsic apoptotic signaling pathway biological process. The label of each vertex has the name of the gene or protein as well as the code of the tissue in which this gene/protein is expressed (L - lymph node, En - endometrium, Em - embryo, R - retina, Sn - substantia nigra)

Additional file 2: Table S2 features information regarding the centrality of the gene network vertices for the analyzed tissues. It appeared that the highest value for betweenness centrality in the gene networks reconstructed for the endometrium, embryo and substantia nigra had the BCL2 protein, in the combined and lymph node networks, the TNF protein, while in the retina network, it was Caspase-3. As such, in the considered networks, the highest centrality vertices presented with different genes/proteins, and it was intriguing to assess the connectivity of these nodes with one another. For this purpose, a new gene network was reconstructed with ANDSystem, which included all three central vertices, i.e., BCL2, TNF and Caspase-3 (Fig. 4). Additionally, vertices intermediaries were included in the network, i.e., nodes interacting with at least two of the three central vertices. It is clearly evident from Fig. 4 that the obtained network is strongly connected. An analysis of this gene network using the FunGeneNet system (http://www-bionet.sscc.ru/fungenenet/) showed that the *p*-value for the significance of the difference of this network from random networks by the number of connections was less than 10^− 11^. In this regard, it can be assumed that the three central vertices of the gene network are represented in a certain functional module and the transition from one central node to another, depending on the tissue, does not lead to a disruption of the apoptosis regulation function.

Table 2 lists the paired correlations between the values of the betweenness centrality for the same genes/proteins, obtained from the networks reconstructed for the analyzed tissues and from the combined network. In cases when the vertex was absent in at least one of the networks, its betweenness centrality value was set to zero. The highest correlation was observed for the pair, lymph node and combined network (*R* = 0.985), while the smallest was for retina and combined network (*R* = 0.398). As is observed in Table 2, for some pairs of tissues, the values of the betweenness centrality varied widely. Thus, considering tissue specificity can be important in the analysis of gene networks, in particular, solving the problem of finding the most significant central genes.

**Table 2:** Pairwise correlation between the values of the betweenness centrality of vertices from the reconstructed networks (combined network and tissue networks)

Tissue	Combined network	Lymph node	Endometrium	Embryo	Retina	Substantia nigra
Combined network	1000	0,985	0,804	0,651	0,398	0,788
Lymph node	0,985	1000	0,772	0,633	0,401	0,792
Endometrium	0,804	0,772	1000	0,787	0,483	0,954
Embryo	0,651	0,633	0,787	1000	0,659	0,792
Retina	0,398	0,401	0,483	0,659	1000	0,530
Substantia nigra	0,788	0,792	0,954	0,792	0,530	1000

## Conclusion

Reconstruction of gene networks that describe the biological and pathological processes occurring in a given tissue requires the consideration of tissue-specific gene expression. A new version of ANDSystem, containing information on tissue-specific expression for 272 tissues, provides such a feature. Analysis of the gene network of the extrinsic apoptotic signaling pathway using the new version of ANDSystem has shown that a change in the structure of the gene network can take place when considering different tissues. Regarding the example of the reconstructed gene networks for the five selected tissues herein, it was demonstrated that applying tissue-specificity filtering to their nodes affects such network characteristics as betweenness of centrality of vertices, clustering coefficient, network centralization, network density, etc. Thus, the newly developed version of ANDSystem, which provides an automated reconstruction of gene networks taking into account tissue-specific expression of genes, can find application in a wide range of medical and biological studies aimed at analysis of various biological processes within individual tissues.

## Additional files

Additional file 1: **Table S1.** Lists of genes involved in extrinsic apoptotic signaling pathway expressed in different tissues. (XLSX 187 kb)

Additional file 2: **Table S2.** Values of betweenness centrality for gene network of extrinsic apoptotic signaling pathway filtered according to tissue-specific expression of genes. (XLSX 45 kb)
